# Diagnostic Accuracy of Lateral Flow Blood Tests to Detect Large Vessel Occlusion Stroke

**DOI:** 10.1161/SVIN.125.002233

**Published:** 2026-05-05

**Authors:** Lisa Shaw, Rachel Binks, David Burgess, Anand Dixit, Edoardo Gaude, Clare Lendrem, Graham McClelland, Philip White, Gewei Zhu, Christopher I. Price

**Affiliations:** 1 Stroke Research Group, Population Health Sciences Institute (L.S., G.Z., C.I.P.), Newcastle University, United Kingdom.; 2 Translational and Clinical Research Institute (P.W.), Newcastle University, United Kingdom.; 3 National Institute for Health and Care Research (NIHR) HealthTech Research Centre in Diagnostic and Technology Evaluation, Newcastle University and Newcastle upon Tyne Hospitals National Health Service (NHS) Foundation Trust, United Kingdom (R.B., C.L.).; 4 Newcastle upon Tyne Hospitals NHS Foundation Trust, Freeman Hospital, United Kingdom (A.D.).; 5 Pockit Diagnostics Ltd trading as Upfront Diagnostics, CR UK Cambridge Institute, United Kingdom (E.G.).; 6 Department of Nursing, Midwifery and Health, Faculty of Health and Life Science, Northumbria University, Newcastle upon Tyne, United Kingdom (G.M.).

**Keywords:** biomarkers, emergency medical services, glial fibrillary acidic protein, stroke, thrombectomy

## Abstract

**BACKGROUND:**

A rapid and accurate tool to identify large vessel occlusion stroke (LVO) for use by prehospital emergency medical services responders could support direct access to time-critical thrombectomy at regional Comprehensive Stroke Centers. This study evaluated the accuracy of D-dimer and GFAP (glial fibrillary acidic protein) detected by lateral flow tests (LFT) to identify LVO stroke.

**METHODS:**

This hospital-based prospective observational cohort study recruited adults within 6 hours of onset of at least 1 face, arm, or speech test (FAST) symptom. The LFTs use fingerprick capillary blood and give an overall positive (ie, LVO stroke predicted) or negative LFT outcome. Independent adjudication of brain imaging and clinical data established LVO or non-LVO. Specificity, sensitivity, negative and positive predictive values were calculated for (1) LFT outcome for participants with >1 FAST symptoms (primary analysis population); (2) a combined decision rule where participants with 1 FAST symptom were rule negative, patients with >1 FAST symptoms rule positive or negative according to LFT outcome; (3) FAST symptoms alone split by 1 symptom versus >1 symptom.

**RESULTS:**

The study involved 382 participants (age [mean], 72.4 years; female, 50.8%; National Institutes of Health Stroke Scale score [median], 6; time from onset to LFT [median], 130 minutes). Of 278 participants who met the primary analysis population, 202 of 278 had both LFT outcome and LVO/non-LVO assigned. Analysis gave specificity 79% (95% CI, 72%–85%), sensitivity 53% (95% CI, 39%–67%), negative predictive value 86% (95% CI, 79%–90%), and positive predictive value 42% (95% CI, 30%–55%). For 382 participants with 1 to 3 FAST symptoms, 290 of 382 had both a combined decision rule result and LVO/non-LVO assigned giving specificity 86% (95% CI, 82%–90%), sensitivity 51% (95% CI, 37%–65%), negative predictive value 90% (95% CI, 86%–93%), positive predictive value 42% (95% CI, 30%–55%). FAST symptoms alone (n=318) gave specificity 33% (95% CI, 27%–38%), sensitivity 96% (95% CI, 87%–99%), negative predictive value 98% (95% CI, 92%–99%), positive predictive value 23% (95% CI, 18%–29%).

**CONCLUSIONS:**

LFT outcome combined with FAST symptoms gave high specificity for LVO identification. This approach could be considered for facilitation of direct access to thrombectomy providers. However, further research is required to evaluate test performance in the prehospital setting and consider technological improvements to boost sensitivity without compromising specificity.

**REGISTRATION:**

URL: https://www.isrctn.com/; Unique identifier: ISRCTN12414986.

**CLINICAL PERSPECTIVE**

- A tool for prehospital identification of large vessel occlusion stroke could facilitate direct access to thrombectomy treatment at regional centers.
- This hospital study evaluated lateral flow tests for D-dimer and glial fibrillary acidic protein biomarkers combined with face arm speech test symptoms and demonstrated high specificity for large vessel occlusion identification.
- Further research is now required to evaluate the lateral flow tests in the prehospital setting and to improve sensitivity for large vessel occlusion detection without compromising specificity.

Large vessel occlusion (LVO) ischemic stroke carries a high risk of severe disability and death^1^ but the chances of recovery to independence significantly improve after mechanical thrombectomy.^2,3^ Earlier treatment produces better outcomes^2^ but internationally, rapid access is challenging because the specialist workforce and facilities required are only available in regional comprehensive stroke centers (CSC). Currently, onward transfer from a primary stroke center is required for many mechanical thrombectomy-eligible patients, increasing the time to treatment and resulting in poorer outcomes.^4,5^ For example, in England, United Kingdom, >100 hospitals provide acute stroke care, but only 1 in 4 of these are CSC thrombectomy providers.^6,7^

An alternative approach to facilitate access to mechanical thrombectomy is prehospital identification of suspected stroke patients likely to have LVO for selective redirection towards a CSC.^8^ However, for this option to be feasible, a tool for rapid and accurate LVO recognition is required, which can be used by emergency medical services responders. Although symptom scale scores have been used for this purpose in some international settings,^9^ other countries, including the United Kingdom, have not adopted this approach due to concerns about accuracy. For any identification tool, there is always a trade-off between higher sensitivity (fewer false negative “missed” cases) or higher specificity (fewer false positive “wrong” cases), and preferences may differ according to the context. In the United Kingdom, existing scales with higher sensitivity but lower specificity risk destabilizing stroke services due to high volumes of patients which would be identified for direct CSC admission.^7^ In this context, tools which prioritize specificity are likely to be preferred in order to minimize direct CSC admission of false positive non-LVO patients who could receive all appropriate care at a nearer PSC. For false negative LVO patients who arrive at a local primary stroke center due to lower sensitivity, thrombectomy can still be accessed using the existing transfer pathway.

Previous work from our team evaluated venous blood samples from suspected stroke patients and identified that combining concentrations of D-dimer and GFAP (glial fibrillary acidic protein) with face arm speech test (FAST) symptoms generated an area under the receiver operating characteristic curve of 93% (95% CI, 90–98), which gave a prioritized specificity of 93% (95% CI, 86–97) and sensitivity of 78% (95% CI, 56–93) for LVO prediction.^10^ D-dimer is a breakdown product of fibrin positively associated with LVO,^11^ whereas GFAP is released following injury of glial cells and is more likely to be elevated in hemorrhagic stroke.^12^ The promising performance of these biomarkers led to the development of portable lateral flow tests (LFT) for detection in fingerprick capillary samples, with results available within 10 to 15 minutes (LVOne test, Pockit diagnostics Ltd, Upfront DX, United Kingdom). Separate LFTs detect D-dimer and GFAP, which are read together to give an overall positive (ie, LVO stroke predicted) or negative LFT outcome.

The aim of this study was to prospectively evaluate the accuracy of the LFT outcome to identify LVO stroke in patients presenting with FAST symptoms and within 6 hours of suspected stroke onset.

## Methods

### Design

A prospective observational cohort study was performed. The full protocol has previously been published.^13^ Reporting follows STARD guidelines (Standards for Reporting of Diagnostic Accuracy Studies).^14^ Ethics approval was granted by the Newcastle and North Tyneside 2 Research Ethics Committee (reference 23/NE/0043). Data from this study will not be available for sharing.

### Setting

Although the future intended use of the LFTs is the prehospital setting, this first-ever real-world evaluation was undertaken by hospitals receiving suspected stroke admissions from emergency medical services responders (ambulance practitioners). Six hospitals in England hosted the study, and trained hospital staff performed the LFTs immediately after eligible patients arrived at hospital.

### Participants

Eligibility for inclusion in this study was assessed by hospital stroke staff during the routine urgent clinical review, which occurs immediately following ambulance arrival with a patient with suspected stroke. To be included patients had to: arrive at hospital by ambulance with the attending ambulance practitioner suspecting a new acute stroke, be ≥18 years, have a least 1 FAST symptom present (ie, FAST score 1–3 points), be within 6 hours of stroke symptom onset or last know well time, and be able to have blood sampling for the LFTs undertaken before any reperfusion treatment. Patients were excluded if attendance was because of a transfer for continuing care or if there had been a recent (<4 weeks) diagnosis of a health event which could result in elevated D-dimer or GFAP including deep vein thrombosis, pulmonary embolism, arterial embolism, stroke, transient ischemic attack, long bone fracture, major trauma, head injury, or any surgery under general anesthesia.

Approach for study enrollment and consent was conducted after all emergency assessments (including the LFTs) and treatments were completed to avoid delays to time-critical stroke care. A range of consent options were available to cover capacity, communication, and discharge statuses, which included individual, personal consultee, professional consultee, and postal versions of information.

### Lateral Flow Tests

**Figure 1:**
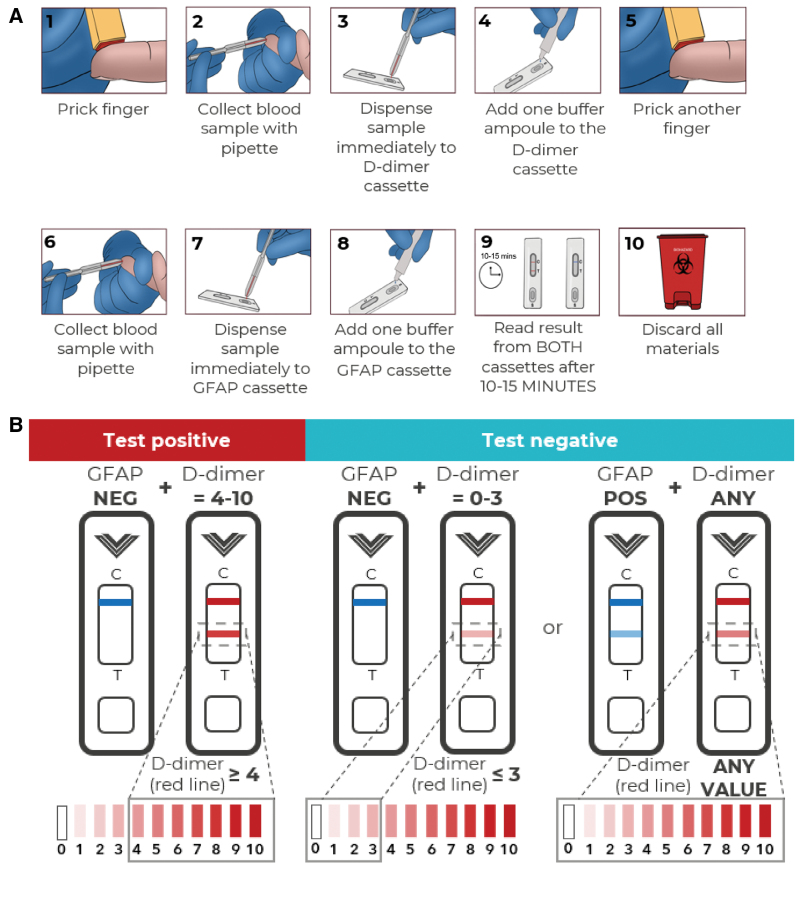
. **Lateral flow testing process and interpretation.** Lateral flow testing process (**A**) and interpretation (**B**). GFAP indicates glial fibrillary acidic protein; NEG, negative; and POS, positive.

Separate lateral flow cassettes detect D-dimer and GFAP (Pockit Diagnostics Ltd, Upfront DX, United Kingdom). A capillary blood drop is pipetted onto each cassette from a fingerprick site, and a buffer solution is added (Figure 1A). Tests are read by the clinician after 10 to 15 minutes. If a test line appears on the D-dimer assay, this is graded visually from 1 to 10 using a score card. For the GFAP assay, only the presence or absence of a test line is read. An overall positive LFT outcome (ie, LVO stroke predicted) is defined as a present D-dimer test line with intensity score ≥ 4 (blood concentration ≥600 ng/mL) with an absent GFAP test line (blood concentration <213 pg/mL).^10,15^ All other test line combinations are considered to represent a negative LFT outcome (ie, non-LVO predicted; Figure 1B).

### Reference Standard

All participants were assigned one of the following clinical outcome states: ischemic stroke with LVO, ischemic stroke without LVO, ischemic stroke with unknown LVO status, intracerebral hemorrhage, transient ischemic attack, mimic condition, or no outcome state can be assigned.

Clinical outcome states were assigned by an independent stroke specialist after review of imaging findings reported by an independent neuroradiologist and participant clinical information (clinical symptoms, observations, previous medical history, medication, laboratory blood test results, and diagnosis provided by a local site stroke clinician). Both the stroke specialist and neuroradiologist were blinded to the LFT results, and the radiologist was also blinded to clinical information. If the independent stroke specialist was unable to assign an outcome, for example, due to discrepancies between the local stroke clinician's diagnosis, clinical information, or independent radiology reports, the case was discussed with a diagnostic adjudication committee consisting of 3 expert stroke clinicians.

Clinical outcome states were formally defined as shown in Table S1 and Figure S1 shows the assignment decision tree. For ischemic stroke with LVO to be the clinical outcome, computed tomography angiography (CTA) or magnetic resonance angiography (MRA) must have been conducted, and an occlusion seen in any large branch of the anterior (intracranial internal carotid artery, M1, M2, or anterior cerebral artery) or posterior (any basilar or vertebral) cerebral circulation. For ischemic stroke without LVO, no such occlusion was seen on CTA/MRA, combined with a local site clinician diagnosis of ischemic stroke. If the local clinician provided a diagnosis of ischemic stroke but CTA/MRA had not been conducted, the clinical outcome assigned was ischemic stroke with unknown LVO status.

For analyses, all clinical outcome states that were not ischemic stroke with LVO were considered non-LVO, except for cases of ischemic stroke with unknown LVO status and those where no outcome state could be assigned. These participants were not included in diagnostic accuracy calculations because they could not be considered to be either LVO or non-LVO.

### Sample Size

The study was powered to detect test specificity, and the calculation was based on a primary analysis population comprising patients with >1 FAST symptoms (ie, FAST score 2 or 3) who were within 6 hours of onset; ie, the calculation did not consider FAST 1 patients. This primary analysis population was chosen for the power calculation because it reflects the most likely future deployment of the LFTs. However, the study overall recruited a broader population to allow for additional analyses (see below).

For the primary analysis population, using an estimated LVO prevalence of 32%, minimum specificity of 80%, *α*=0.05, and *β*=0.1, 123 participants were required to detect a specificity of 92% for LVO versus all non-LVO outcomes. To account for missing LFT results or inability to categorize cases as LVO or non-LVO (predominantly expected to be because CTA/MRA had not been conducted), the required sample size for the primary analysis population was inflated to an estimated 276 cases and study management processes involved regular monitoring of available data such that the study ceased once it was clear that the required sets of data had been obtained. As the broader study population would be enrolled in parallel with the primary population, a higher total number of patients was expected.

### Analyses

Using the primary analysis population, the diagnostic accuracy (specificity, sensitivity, negative predictive value, positive predictive value) of the LFT outcome for identification of LVO stroke was determined. Confidence intervals were calculated using the Agresti-Coull method.^16^

The population comprising cases with any FAST symptoms (ie, score 1, 2, or 3) and within 6 hours of symptom onset (FAST population) was used to determine the diagnostic accuracy of the following:

1. A combined decision rule where patients with 1 FAST symptom were labeled as rule negative irrespective of the LFT outcome, whereas patients with >1 FAST symptoms (ie, 2 or 3 FAST symptoms) were labeled rule positive or negative according to the LFT outcome.^15^
2. The FAST score in isolation for LVO detection at a split of 1 (test negative) versus >1 symptom (test positive).

Specificity, sensitivity, negative and positive predictive values are reported with CIs calculated using the Agresti-Coull method.^16^

## Results

Participants were enrolled in the study between August 25, 2023, and August 20, 2024. A total of 278 participants fulfilled the primary analysis population criteria (ie, FAST 2 or 3 and within 6 hours of symptom onset), and 382 the broader FAST population (ie, ≥1 FAST symptoms and within 6 hours of symptom onset). Participant characteristics are shown in Table 1. The clinical outcome states were: 242 of 382 (63.4%) ischemic stroke of which 54 of 242 (22.3%) were LVO, 128 of 242 (52.9%) non-LVO, 60 of 242 (24.8%) ischemic stroke with unknown LVO status; 51 of 382 (13.4%) intracerebral hemorrhage; 19 of 382 (5.0%) transient ischemic attack; 66 of 382 (17.3%) mimic condition and 4 of 382 (1.0%) no outcome could be assigned. For the 54 LVO cases, 48 of 54 (89%) were anterior LVO and 6 of 54 (11%) were posterior LVO. The time to LFT test from hospital arrival was a median of 11 minutes (interquartile range, 6–24) and from symptom onset, median 130 minutes (interquartile range, 95–179).

**Table 1.**
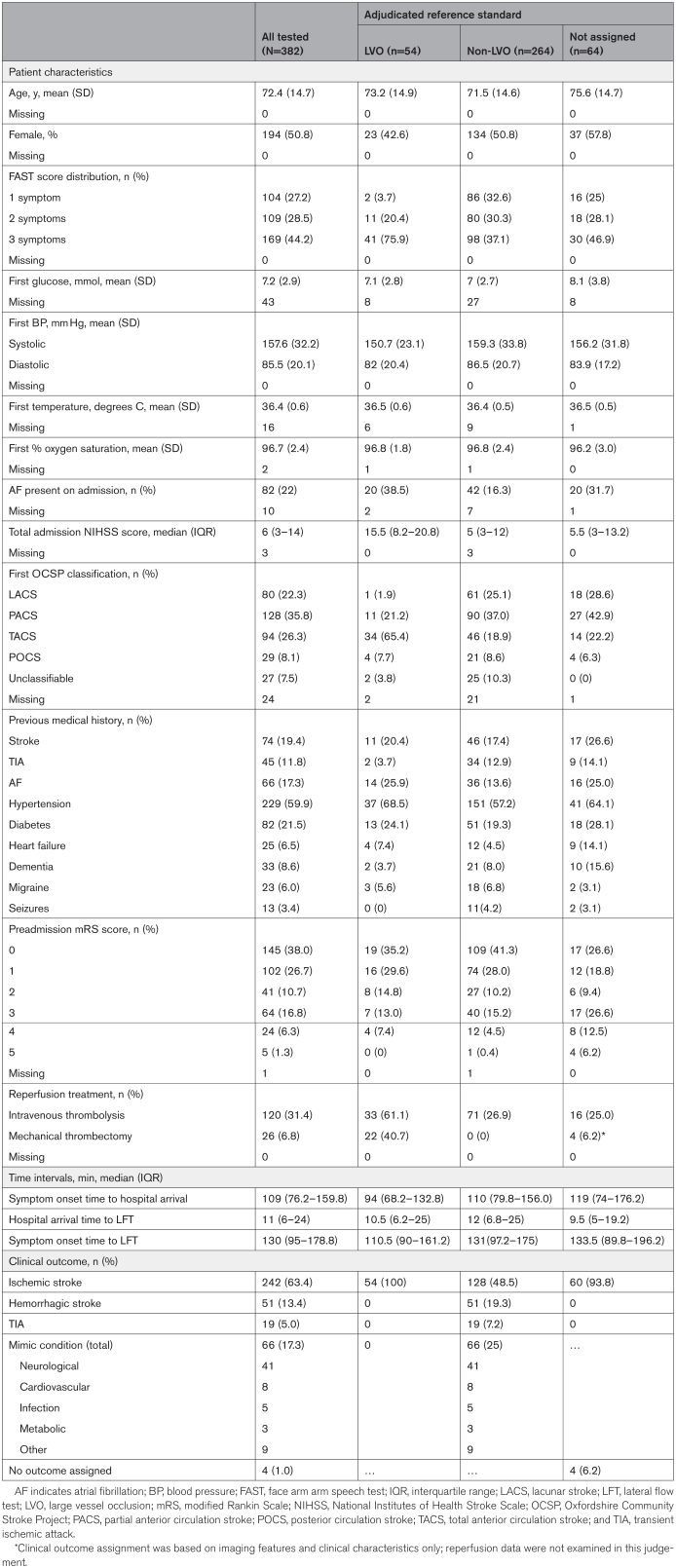
Participant Characteristics

**Figure 2.**
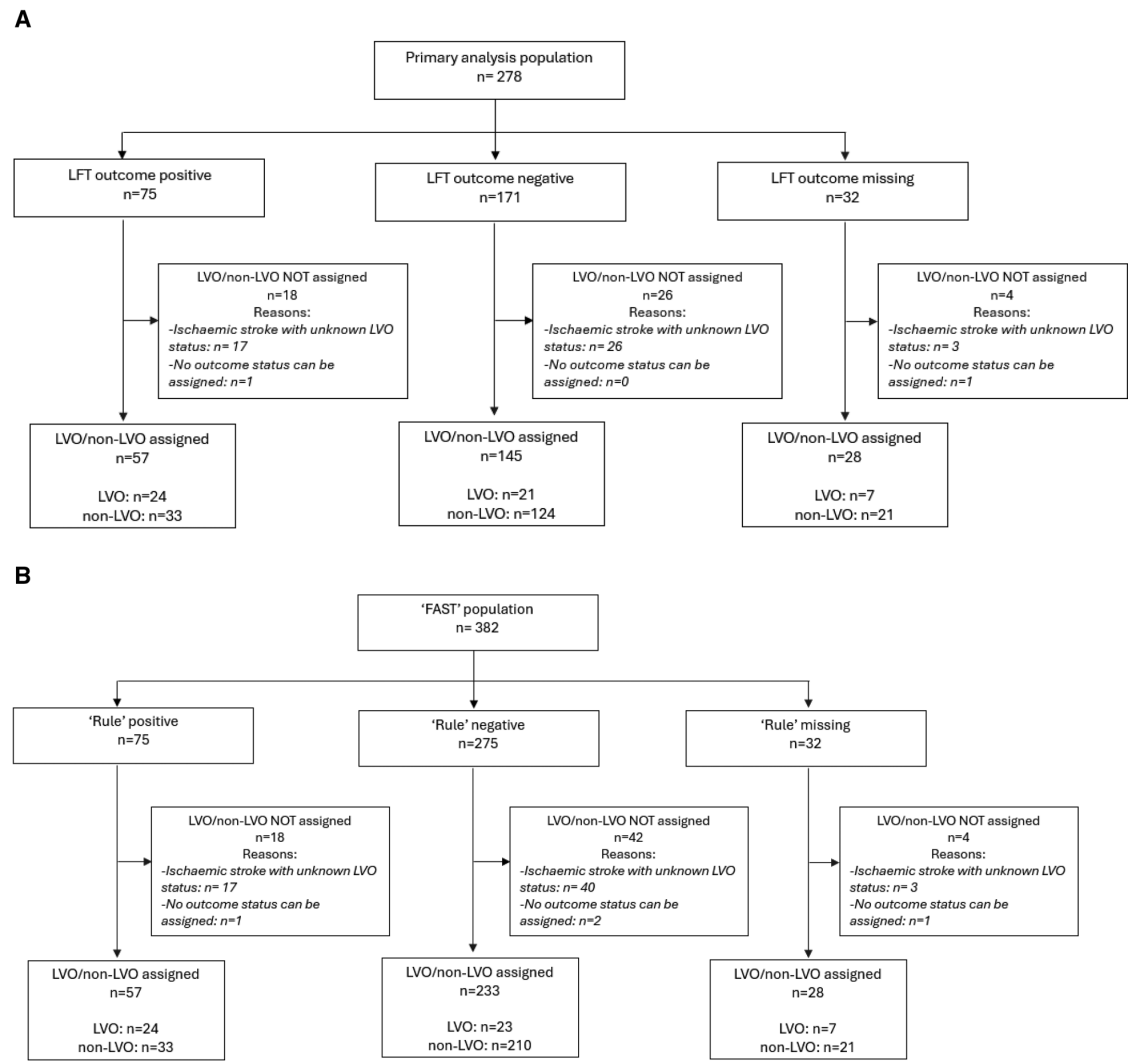
**Study flow diagrams.** Study flow diagrams for primary analysis population (**A**) and FAST population (**B**). FAST indicates face, arm, speech test; LFT, lateral flow test; and LVO, large vessel occlusion.

Considering the primary analysis population, 202 of 278 participants had both a LFT outcome and LVO/non-LVO status assigned (Figure 2A). The prevalence of LVO was 22% (45/202). For the FAST population, 290 of 382 participants had both a combined decision rule result and LVO/non-LVO status assigned (Figure 2B), and the prevalence of LVO was 16% (47/290). LFT outcomes were unavailable due to: fingerprick undertaken but blood not transferred to lateral flow cassette (n=2), control line not visible, which rendered the test void (n=28), test information missing from the study data set (n=2). LVO/non-LVO status was unavailable predominantly due to ischemic stroke cases that did not undergo CTA/MRA.LVO identification results are shown in Table 2. In the primary analysis population, the LFT outcome demonstrated specificity of 79% (95% CI, 72–85) and sensitivity of 53% (95% CI, 39–67). The combined decision rule generated a specificity of 86% (95% CI, 82–90) with a sensitivity of 51% (95% CI, 37–65). When the FAST score was used by itself to predict LVO, with >1 FAST symptoms being considered a positive test, the sensitivity was much increased at 96% (95% CI: 87–99), but at the cost of considerably poorer specificity of 33% (95% CI, 27–38).Patient profiles associated with incorrect identification using the combined decision rule are shown in Table 3. Of the 33 cases with false positive rule results, 17 of 33 (51.1%) were ischemic stroke without LVO, 6 of 33 (18.2%) were intracerebral hemorrhage, 2 of 66 (6.1%) transient ischemic attack, and the remaining 8 of 33 (24.2%) were nonstroke mimic conditions. Of the cases with false negative rule results, only 2 had just 1 FAST symptom. Otherwise, the median National Institutes of Health Stroke Scale score and the presence of/history of Atrial Fibrillation were similar to the true positive cases.

**Table 2.**
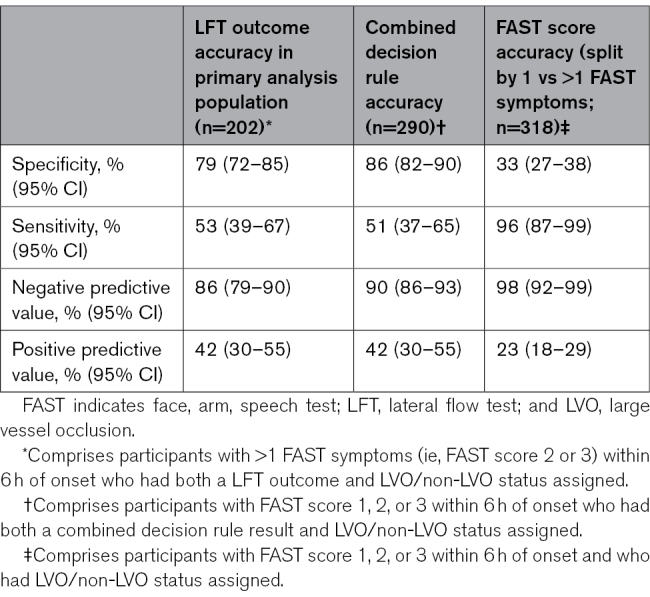
LVO Identification Performance

**Table 3.**
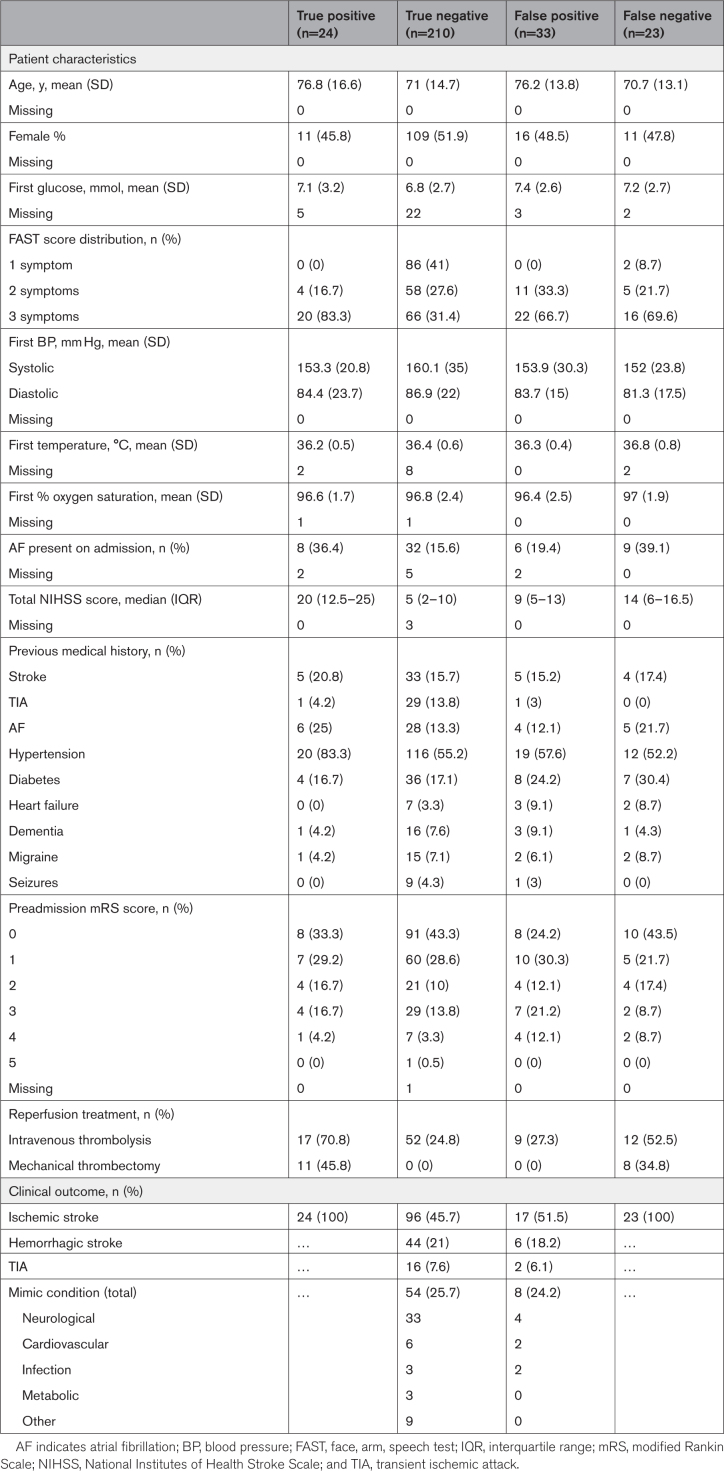
Participant Characteristics According to the Combined Decision Rule Performance

No complications were reported from fingerprick sampling.

## Discussion

This is the first published description of capillary blood lateral flow testing being used during emergency stroke assessment to identify LVO. When LFT readings were obtained from suspected stroke patients with 2 or 3 FAST symptoms and within 6 hours of onset, specificity for LVO identification was 79%. When LFT readings were incorporated with FAST symptoms into a combined decision rule, specificity was increased to 86%. In both cases, approximately half of the participants with LVO stroke were correctly identified. The diagnostic accuracy was superior to using FAST scores alone (split of 1 [test negative] versus >1 symptom [test positive]), where a specificity of only 33% was obtained with a positive predictive value of only 23%.

As described previously, development of the LFTs followed a venous blood sample evaluation using laboratory measurement of D-dimer and GFAP, where a specificity of 93% and sensitivity of 78% could be obtained.^10^ Since starting our study, a second cohort of venous samples with laboratory measurement of D-dimer and GFAP has been reported, where in this case the combined decision rule was assessed, giving a specificity of 92% and sensitivity of 80%.^15^ Furthermore, in a laboratory setting, venous samples have been applied to the developed LFTs, and a specificity of 92% and sensitivity of 75% was obtained when considering a decision rule using FAST-ED (Field Assessment Stroke Triage for Emergency Destination) symptoms (rather than FAST).^17^ While our current study reports poorer accuracy than any of these earlier studies, important differences are that our evaluation used capillary blood sampling for the LFTs, the sampling and LFTs were performed in real world emergency assessment of patients, and results were read by attending clinicians. Differences in accuracy could relate to venous versus capillary biomarker concentrations, clinician assessment of LFT lines, and differences in reference standard definitions of LVO or non-LVO.

The biomarker thresholds in the LFTs were set to maximize specificity over sensitivity, and changes could be made with the aim of improving sensitivity, for example, lowering the D-dimer line intensity for a positive test outcome. However, sensitivity changes should be carefully balanced against specificity, as most CSCs which would receive redirected patients, will wish to minimize non-LVO false positives and would likely be reluctant to adopt a triage technology that is perceived as inefficient. This is due to finite resources at CSCs^7^ and also concerns that non-LVO patients who travel for longer to get to the hospital might experience poorer care that offsets the population-level benefits of accessing thrombectomy more quickly.^18^ In the RACECAT randomized controlled trial (Direct Transfer to an Endovascular Center Compared to Transfer to the Closest Stroke Center in Acute Stroke Patients With Suspected Large Vessel Occlusion), which selected ambulance patients for redirection to CSC using the Rapid Arterial Occlusion Evaluation clinical score, thrombectomy delays were reduced, but there was also a reduction and delays in intravenous thrombolysis treatment for ischemic stroke, and concern was raised about poorer outcomes among hemorrhagic stroke.^18,19^

The combined decision rule used the FAST score rather than any longer or more complex symptom score because FAST is already used routinely by UK ambulance services, and future implementation would avoid the need for additional clinical assessment training. It is possible that combining LFT results with other symptoms strongly associated with LVO, such as gaze deviation, could further improve accuracy; however, the recent venous sampling study reported only a marginal improvement in specificity (92% to 93%) and no change in sensitivity when using FAST-ED rather than FAST in the decision rule.^15^ Introduction of a more sophisticated clinical assessment would require additional training of most emergency medical services workforces.

When considering the use of symptoms alone for identification of LVO, the combined decision rule performed well in comparison to scores constructed in a recent pooled analysis of 2 ambulance data sets.^20^ In this retrospective analysis, the Cincinnati Prehospital Stroke Scale+gaze score achieved a specificity of 94% (95% CI, 93–95), but with a sensitivity of 35% (95% CI, 29–41) and positive predictive value of 40% (95% CI, 33–47); the Emergency Medical Stroke Assessment score achieved a higher sensitivity of 85% (95% CI, 80–90), but with a specificity of only 58% (95% CI, 56–61) and positive predictive value of 18% (95% CI, 16–20). The best balance of overall performance was from the Rapid Arterial Occlusion Evaluation score, which gave a specificity of 86% (95% CI, 84–88) and sensitivity of 64% (95% CI, 58–70), but the positive predictive value was only 33% (95% CI, 29–38). Due to complexity, routine use of the Rapid Arterial Occlusion Evaluation score would create a high training and quality assurance burden for ambulance services, and implementation of the LFTs in combination with FAST is likely to be considered preferable.

Although other portable technologies are under evaluation to identify LVO, such as dry electrode electroencephalogram^21^ and cranial accelerometry,^22^ to our knowledge, no approach has yet been demonstrated as sufficiently accurate or feasible for prehospital use. Although suspected stroke is a common medical emergency at population level, it is not encountered by individual emergency service practitioners on a very frequent basis, so it is important that any test is intuitive to use and interpret, as well as accurate.^23^ Technology requiring unfamiliar equipment is less likely to be used correctly and takes longer to deploy than a LFT, which can develop in parallel with other patient care tests once capillary blood has been applied. However, we acknowledge that further improving the accuracy of the LFT outcome would be beneficial, and this might be achieved by technology improvements, such as reducing the possibility of test line reading errors. For example, the addition of a digital reader smartphone application to detect and correctly interpret the test lines.

In terms of future use of the LFTs, it is important to note that not all patients with LVO stroke are eligible for thrombectomy, and the ultimate aim of prehospital selection for redirection should be to identify thrombectomy candidates. One possibility is that thrombectomy selection could be facilitated by an ambulance practitioner to hospital stroke specialist communication, whereby contact (eg, by telephone or video call) is made about cases with a positive LFT outcome or positive combined decision rule, and additional clinical parameters relevant to a thrombectomy decision are discussed to make the decision about redirection accordingly. Our team has recently commenced an observational study which will evaluate both the performance of the LFT outcome when the tests are used in the prehospital setting and determine the value of a clinical communication step to assess thrombectomy suitability.^24^ Evaluation of the LFTs when used in the prehospital setting is important because deployment and performance could be influenced by multiple factors, including workforce training, patient selection, environmental conditions, and service pressures.

A limitation of this study is that patients were tested shortly after hospital arrival rather than in the prehospital setting. For an initial evaluation of the LFTs, a hospital setting enabled evidence to be generated quickly in a very similar clinical population while avoiding the substantial challenges that arise when conducting a prehospital study, such as the requirement for stocking LFTs in all ambulances, training large numbers of geographically dispersed ambulance personnel, and linking prehospital and hospital patient data. However, it is acknowledged that test accuracy may differ when deployed in the prehospital setting for reasons including different usability challenges and patient selection. A further study limitation is that it was not possible to mandate that patients should have CTA or MRA to confirm LVO status, and such cases had to be excluded from the analyses, potentially producing results that could be less generalizable.

Strengths of the study included the use of multiple options for invitation and consent (eg, options for incapacity, rapid discharge, and death) to attempt to avoid a population bias and a standardized approach to assign clinical outcomes via independent adjudication giving a high likelihood of unbiased and reproducible data.

## Conclusions

In conclusion, combining the LFT outcome with FAST symptoms gave high specificity for LVO stroke identification and this approach could be considered for facilitation of direct access to thrombectomy providers. However, further research is required to evaluate test performance in the prehospital setting and consider technological improvements to boost sensitivity without compromising specificity.

## ARTICLE INFORMATION

### Acknowledgments

The authors thank the patients who participated in the study and the clinical and research teams at the following hospitals: Newcastle on Tyne Hospitals National Health Service (NHS) Foundation Trust, Northumbria Healthcare NHS Foundation Trust, County Durham and Darlington NHS Foundation Trust, East Lancashire Hospitals NHS Trust, Royal Wolverhampton NHS Trust, and St George’s University NHS Foundation Trust.

### Author Contributions

Dr Shaw, D. Burgess, Dr Dixit, Dr Gaude, Dr Lendrem, Dr McClelland, and Prof Price contributed to the funding application and design of the study. Dr Shaw and Prof Price led the development of the study protocol, obtained ethical approval, and led delivery of the study. Drs Lendrem and Binks provided methodological and statistical support. Prof White provided radiological adjudication expertise. Dr Zhu supported the management of the study. Dr Shaw and Prof Price wrote the first draft of the manuscript. All authors reviewed and edited the manuscript and approved the final version.

### Disclosures

The employing institutions of all authors received funds from the Small Business Research Initiative Healthcare (reference: SBRIH18P2003) to enable this work to be undertaken. Dr Shaw reports grants from the National Institute for Health and Care Research (NIHR) and the Small Business Research Initiative outside of the submitted work. Dr Gaude reports a grant from the Small Business Research Initiative Healthcare outside of the submitted work, shares in Pockit diagnostics Ltd, and a granted patent covering biomarkers for large vessel occlusion detection (WO2021209732A1). Dr McClelland reports grants from NIHR and the Small Business Research Initiative outside of the submitted work. Dr White reports grants from the NIHR outside of the submitted work. Dr Price reports grants from NIHR and the Small Business Research Initiative outside of the submitted work. The other authors report no conflicts.

### Supplemental Material

Table S1

Figure S1

## Supplementary Material
