# Supplementary material for: Diagnostic Accuracy of Lateral Flow Blood Tests to Detect Large Vessel Occlusion Stroke
Source: Stroke Vasc Interv Neurol. 2026 May 5;6(3):e002233. doi: 10.1161/SVIN.125.002233 (PMC13138473; doi:10.1161/SVIN.125.002233)

SUPPLEMENTARY MATERIAL

Diagnostic Accuracy of Lateral Flow Blood Tests to Detect Large Vessel Occlusion Stroke

Lisa Shaw[1], Rachel Binks[2], David Burgess[3], Anand Dixit[4], Edoardo Gaude[5], Clare Lendrem[2], Graham McClelland[,6], Philip White[7], Gewei Zhu[1], Christopher I. Price[1]

1. Stroke Research Group, Population Health Sciences Institute, Newcastle University, Henry Wellcome Building, Newcastle Upon Tyne, NE2 4HH, UK.

2. NIHR HealthTech Research Centre in Diagnostic and Technology evaluation, Newcastle University and Newcastle upon Tyne Hospitals NHS Foundation Trust, The Medical School, Newcastle upon Tyne, NE2 4HH.

3. Service user representative. Contact via: Stroke Research Group, Population Health Sciences Institute, Newcastle University, Henry Wellcome Building, Newcastle Upon Tyne, NE2 4HH, UK.

4. Newcastle upon Tyne Hospitals NHS Foundation Trust, Freeman Hospital, Freeman Road, High Heaton, Newcastle upon Tyne, NE7 7DN, UK.

5. Pockit Diagnostics Ltd trading as Upfront Diagnostics, CRUK Cambridge Institute, Robinson way, Cambridge, CB2 0RE, UK.

6. Department of Nursing, Midwifery and Health, Faculty of Health and Life Science, Northumbria University, Newcastle upon Tyne, NE7 7YT, UK.

7. Stroke Research Group, Translational and Clinical Research Institute, Newcastle University, Henry Wellcome Building, Newcastle Upon Tyne, NE2 4HH, UK

Table S1: Clinical outcome state definitions

Outcome state	Definition
Intracerebral haemorrhage	Blinded neuroradiologist recorded haematoma present on CT (and/or MRI).
Ischaemic stroke with large vessel occlusion	CT or MR angiography was conducted and demonstrated reduced filling in any large branch of the anterior or posterior cerebral circulation as recorded by the blinded neuroradiologist.<br>For the anterior circulation, the Ten Point Clot Burden Score <10 indicated LVO. For the posterior circulation, any present basilar artery or acute vertebral artery occlusion indicated LVO.
Ischaemic stroke without large vessel occlusion	Expert local site clinician diagnosis was ischaemic stroke at 72 hours after hospital admission (or discharge/death if sooner) and CT/MR angiography (read by blinded neuro-radiologist) confirmed the absence of LVO (i.e. Clot Burden Score = 10, no basilar artery or acute vertebral artery occlusion).
Ischaemic stroke with unknown LVO status	Expert local site clinician diagnosis was ischaemic stroke at 72 hours after hospital admission (or discharge/death if sooner) and CT/MR angiography was NOT conducted.
Transient ischaemic attack	Expert local site clinician diagnosis was TIA at 72 hours after hospital admission (or discharge/death if sooner) and brain imaging findings (read by blinded neuro-radiologist) did not refute the clinician opinion or indicate the presence of one of the other outcome states.
Mimic condition	Expert local site clinician diagnosis was a mimic condition at 72 hours after hospital admission (or discharge/death if sooner) and brain imaging findings (read by blinded neuro-radiologist) did not refute the clinician opinion or indicate the presence of one of the other outcome states.

Figure S1: Decision tree for assigning a clinical outcome state

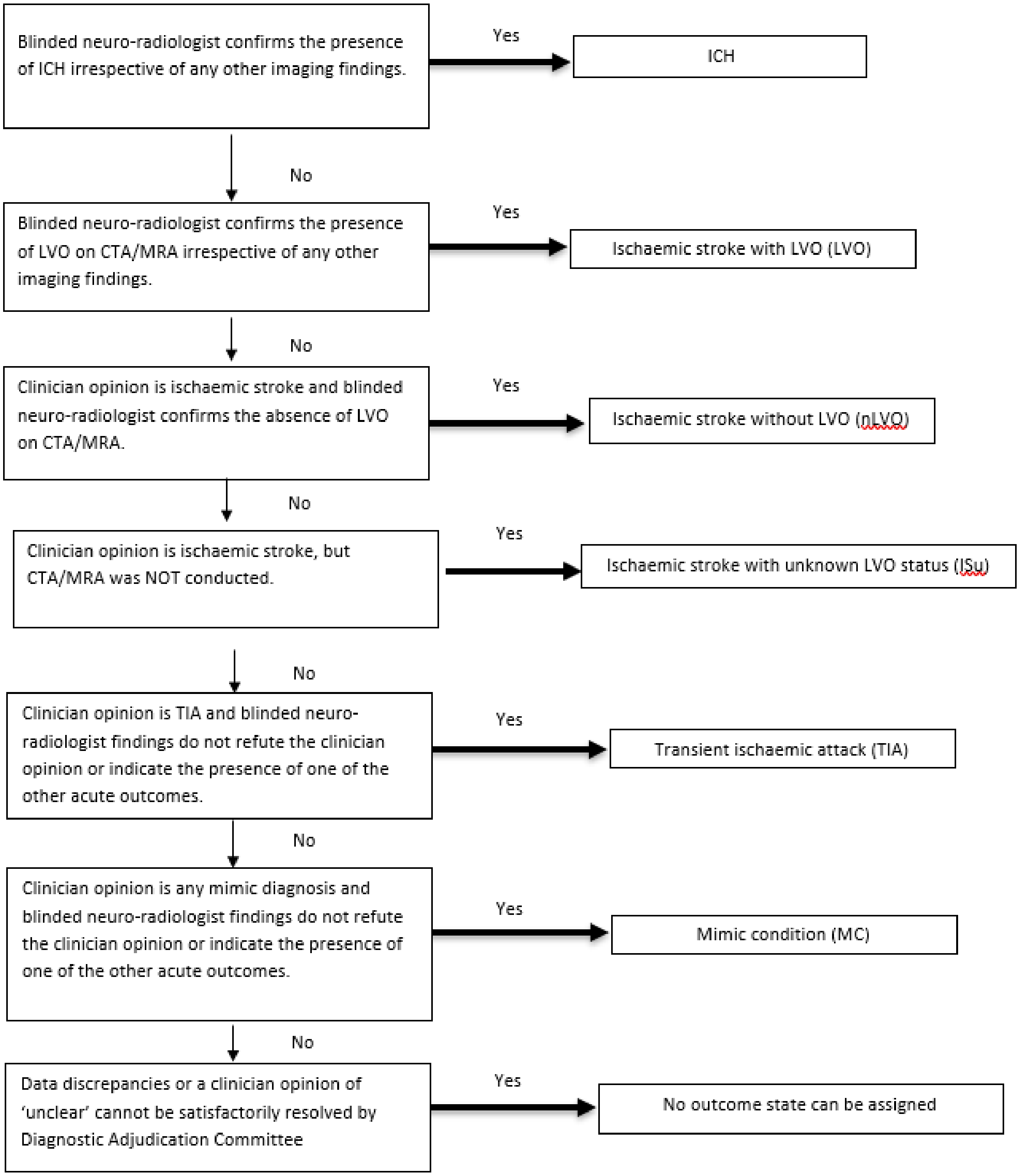

Supplement: Supplementary file 1 [file svi2-6-e002233-s001.pdf]
